# Predicting mortality in critically ill patients: a machine learning approach to electrolyte imbalances and clinical risk factors

**DOI:** 10.1186/s12967-025-07311-7

**Published:** 2025-12-17

**Authors:** Hesham Kamal Habeeb Keryakos, Walid Taha Hussein, Mostafa Ahmed El-Sayed Abu-El-Ela, Aml Kamal Helmy

**Affiliations:** 1 Department of Internal Medicine, Faculty of Medicine, Minia University, Minya, Egypt; 2 Department of Clinical Pathology, Faculty of Medicine, Minia University, Minya, Egypt

**Keywords:** Critical care, ICU mortality, Machine learning, Risk stratification, SOFA score, Metabolic derangements

## Abstract

The study aimed to enhance ICU mortality prediction by integrating **electrolyte imbalances** and **clinical risk factors** using machine learning, addressing gaps in traditional severity scores like SOFA and APACHE II.

**Dominant Predictors of Mortality**: SOFA score ≥ 8 (aOR = 4.0), mechanical ventilation (aOR = 3.1), hyperlactatemia (> 4 mmol/L; aOR = 2.6), vasopressor use (aOR = 2.7), and GCS ≤ 8 (aOR = 2.4).

**High-Risk Phenotypes**: “Triad of Death” (SOFA ≥ 8 + mechanical ventilation + vasopressors), metabolic crisis (lactate > 4 mmol/L + pH < 7.2), and frail elderly (age ≥ 65 with comorbidities).

The **XGBoost model** achieved superior discrimination (**AUC-ROC = 0.86**) compared to logistic regression (AUC = 0.84), SOFA (AUC = 0.72), and APACHE II (AUC = 0.75). A **nomogram** was developed to stratify mortality risk into four actionable tiers (e.g., scores > 150 predicted > 60% mortality).

The model performed consistently across subgroups (sepsis, age strata, AKI stages), with AUCs > 0.80. **Sepsis patients**: Lactate and vasopressor use were stronger predictors (38% contribution to SHAP values)

Early correction of modifiable factors (e.g., sepsis, acidosis) may improve outcomes. The nomogram offers a **practical bedside tool** for risk stratification and clinical decision-making.

## Abstract (structured)

**Background:**

Critically ill patients in intensive care units (ICUs) exhibit high mortality due to complex interactions among metabolic instability, organ dysfunction, and therapeutic interventions. While conventional severity scores like SOFA and APACHE II are widely used, they insufficiently integrate dynamic metabolic markers.

**Methods:**

We performed a retrospective cohort study of 1,004 adult ICU patients. Demographic, clinical, and laboratory data were analyzed using multivariate logistic regression and machine learning algorithms (Random Forest, XGBoost). Model performance was assessed using AUC-ROC, calibration slope, and sensitivity at fixed specificity threshold.

**Results:**

The ICU mortality was 30.7%. Independent predictors of mortality included SOFA score ≥ 8 (aOR = 4.0), mechanical ventilation (aOR = 3.1), hyperlactatemia ≥ 4 mmol/L (aOR = 2.6), vasopressor use (aOR = 2.7), and GCS ≤ 8 (aOR = 2.4). The XGBoost model achieved superior discrimination (AUC = 0.86; 95% CI: 0.83–0.89) compared to logistic regression (AUC = 0.84), SOFA (AUC = 0.72), and APACHE II (AUC = 0.75). A validated nomogram incorporating eight key predictors stratified mortality risk into four actionable tiers. Subgroup analysis confirmed robust performance across age strata, sepsis status, and AKI stages.

**Conclusions:**

Integrating metabolic markers with clinical severity scores enhances ICU mortality prediction. Our nomogram offers a practical, interpretable tool for bedside risk stratification and clinical decision support.

## Key Contributions

•**Mechanistic Insight**: Electrolyte disturbances (e.g., hypomagnesemia) impact mortality primarily through organ dysfunction pathways.
•**Practical Tool**: The nomogram integrates metabolic and clinical parameters for real-time risk assessment.
•**Advancement in Prognostics**: Combines machine learning with interpretable clinical thresholds to bridge gaps in traditional scoring systems.

## Introduction

Critically ill patients admitted to intensive care units (ICUs) represent a complex population with high mortality rates, often exceeding 30% in contemporary studies [1]. These patients frequently present with multi-organ dysfunction syndrome (MODS), severe systemic infections, and profound metabolic disturbances that collectively contribute to poor outcomes [2]. The pathophysiological interplay between these factors creates a clinical challenge for prognostication and targeted intervention. Electrolyte imbalances have emerged as particularly salient markers of illness severity in this population. Hyponatremia (present in 15–30% of ICU admissions) and hyperkalemia (occurring in 5–10% of cases) are not merely laboratory abnormalities but reflect underlying systemic dysfunction [3, 4]. These disturbances arise from multiple mechanisms including neurohormonal activation, renal impairment, and iatrogenic factors, with growing evidence suggesting they independently predict mortality [5]. For instance, a recent meta-analysis demonstrated that even mild hyponatremia (< 135 mEq/L) increases mortality risk by 1.5-2.5-fold across diverse ICU populations [6].

The clinical landscape of critical illness has evolved significantly in recent years, with changing patterns of antimicrobial resistance, advances in organ support technologies, and an aging population with complex comorbidities. Contemporary ICU cohorts now demonstrate higher prevalence of conditions like sepsis-associated AKI (present in 40–50% of septic patients) and multi-drug-resistant infections [7, 8]. These epidemiological shifts necessitate updated risk stratification approaches that account for both traditional severity markers and emerging prognostic factors. Moreover, machine learning (ML) models have emerged as powerful tools in critical care, offering improved predictive performance by leveraging nonlinear interactions among diverse variables. However, their use in clinical practice has been constrained by concerns about interpretability and generalizability.

Current prognostic tools such as the Sequential Organ Failure Assessment (SOFA) and Acute Physiology and Chronic Health Evaluation (APACHE) II scores provide valuable mortality risk estimation [9, 10]. However, these systems have notable limitations in their ability to incorporate dynamic metabolic parameters. The SOFA score, while excellent for assessing organ dysfunction, lacks granularity in evaluating electrolyte disturbances [11]. Similarly, APACHE II includes some metabolic parameters but uses fixed time-point assessments that may miss clinically significant fluctuations [12]. This gap in prognostic modeling becomes particularly relevant given that electrolyte abnormalities often precede overt organ failure by 24–48 h, representing a potential window for intervention [11].

This study addresses three crucial gaps in ICU prognostication:

1. The independent contribution of electrolyte disturbances to mortality after adjusting for organ dysfunction and therapeutic interventions remains unclear [13]. We hypothesize that concurrent electrolyte abnormalities (e.g., combined hyponatremia and hyperkalemia) may exert synergistic mortality effects beyond their individual impacts.
2. Existing severity scores have limited bedside utility due to complex calculations, creating a need for simplified tools that integrate both physiological and metabolic parameters.
3. Current prognostic systems often fail to identify actionable targets; we therefore investigate whether early electrolyte correction independently improves outcomes beyond standard interventions.

To address these gaps, our study pursues three key objectives:

1. Employ advanced multivariate modeling to quantify associations between electrolyte patterns, organ dysfunction markers, therapeutic interventions, and mortality.
2. Develop and validate a pragmatic risk-stratification tool combining severity scores with metabolic parameters for real-time clinical application.
3. Identify modifiable risk factors, particularly electrolyte disturbances, as potential quality improvement targets.

By achieving these aims, this work will provide both mechanistic insights into electrolyte-mediated pathophysiology and practical tools for risk prediction, ultimately enabling earlier identification of high-risk patients and more targeted management of modifiable factors in critical care.

## Subjects and methods

### Study design and setting

This retrospective cohort study was conducted at a tertiary care academic medical center (32-bed medical intensive care unit at Minia University Hospital). We reviewed electronic medical records for all adult patients (≥ 18 years) admitted to the ICU between January 2019 and December 2022. The study protocol was approved by the institutional review board, with a waiver of informed consent due to its observational nature.

### Patient selection

**Fig. 1:**
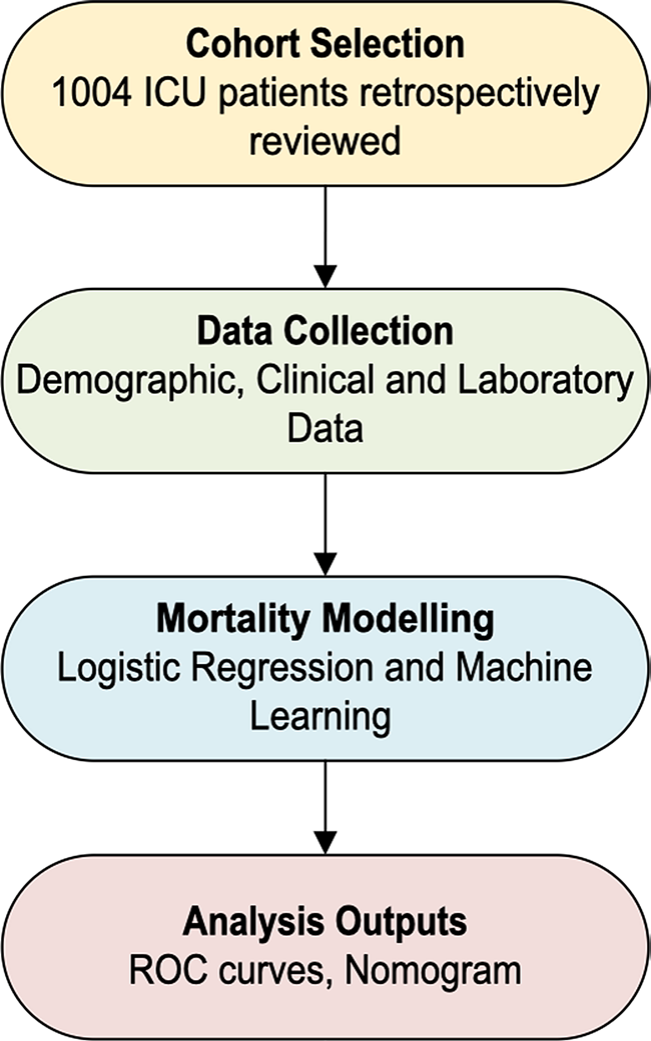
Study design flowchart

Inclusion criteria were: (1) age ≥ 18 years, and (2) ICU stay of at least 24 h. We excluded patients with incomplete clinical or laboratory data (>20% missing data), those transferred from other institutions, and readmissions during the study period (where only the first admission was analyzed). From an initial pool of 1,287 eligible patients, we excluded 283 (22.0%) due to incomplete data, resulting in a final analytical cohort of 1,004 patients (Fig. 1).

### Data collection

We extracted demographic data (age, sex), comorbidities, ICU admission diagnoses such as sepsis, pneumonia, acute kidney injury, and stroke. Documented critical care interventions comprised mechanical ventilation, vasopressor use, and renal replacement therapy (RRT). Laboratory parameters encompassed electrolytes, lactate, creatinine, and hemoglobin levels. Standard severity scores (SOFA, APACHE II, SAPS II) were calculated within the first 24 h of ICU admission using established protocols. Acute kidney injury (AKI) was classified according to KDIGO guidelines.

### Outcome

The primary outcome measure was all-cause ICU mortality.

### Statistical analysis

Data were analyzed using SPSS v28 (Statistical Package for the Social Sciences), with statistical significance defined as two-sided *p* < 0.05. Our statistical methodology employed a multi-tiered analytical approach. Initial descriptive analyses characterized the study population, with categorical variables reported as frequencies and percentages, and continuous variables presented as mean ± standard deviation (SD) for normally distributed data or median with interquartile range (IQR) for non-normally distributed variables. Comparative analyses between survivors and non-survivors utilized independent samples t-tests for normally distributed continuous variables, Mann-Whitney U tests for non-normally distributed continuous variables, and chi-square tests for categorical variables.

### Model development

We implemented a sequential analytical strategy beginning with univariate logistic regression to identify candidate predictors (*p* < 0.05 threshold), followed by multivariate logistic regression with backward stepwise selection while adjusting for key confounders including age, sex, and comorbidities. Model validation procedures included assessment of multicollinearity (variance inflation factor < 5), goodness-of-fit evaluation using the Hosmer-Lemeshow test, and discrimination analysis using area under the receiver operating characteristic curve (AUC-ROC). To enhance the robustness of our findings, we complemented traditional regression approaches with machine learning techniques including Random Forest and XGBoost algorithms, incorporating SHAP values for improved model interpretability.

### Model evaluation

To rigorously assess the model’s generalizability across clinically distinct populations, we performed pre-specified subgroup analyses using stratified sampling and interaction testing. The subgroups were defined a *priori* based on three key clinical dimensions: (1) sepsis status (Sepsis-3 criteria) (2), age strata (< 50, 50–65, and >65 years to capture frailty transitions), and (3) renal function (AKI Stage 2–3 by KDIGO criteria versus non-AKI patients). These subgroups were selected to reflect both pathophysiological heterogeneity (e.g., sepsis versus non-sepsis physiology) and clinically meaningful risk stratification thresholds routinely used in ICU decision-making. For each subgroup, we computed stratified performance metrics including AUC-ROC, sensitivity at 90% specificity, and calibration parameters (slope and intercept). To formally test for effect modification, we incorporated interaction terms (e.g., SOFA × sepsis_status) in multivariate models and used DeLong’s test to compare AUC differences between subgroups. Model fairness was assessed using two complementary approaches: quantitative disparity metrics (equalized odds difference and demographic parity for age/sex subgroups) and qualitative interpretation of SHAP value distributions to identify shifts in feature importance across populations. This dual approach ensured both statistical rigor and clinical interpretability of subgroup differences.

All analyses were conducted on a hold-out test set (30% of the cohort) to mitigate overfitting, with multiple testing correction via the Holm-Bonferroni method. Computational reproducibility was maintained through version-controlled scripts (Python 3.9/R 4.2) that automated subgroup stratification, metric calculation, and visualization. This systematic framework not only validates the model’s robustness across critical subpopulations but also identifies contexts where recalibration may be warranted (e.g., age-specific probability thresholds).

## Results

### Demographic and clinical characteristics

**Fig. 2:**
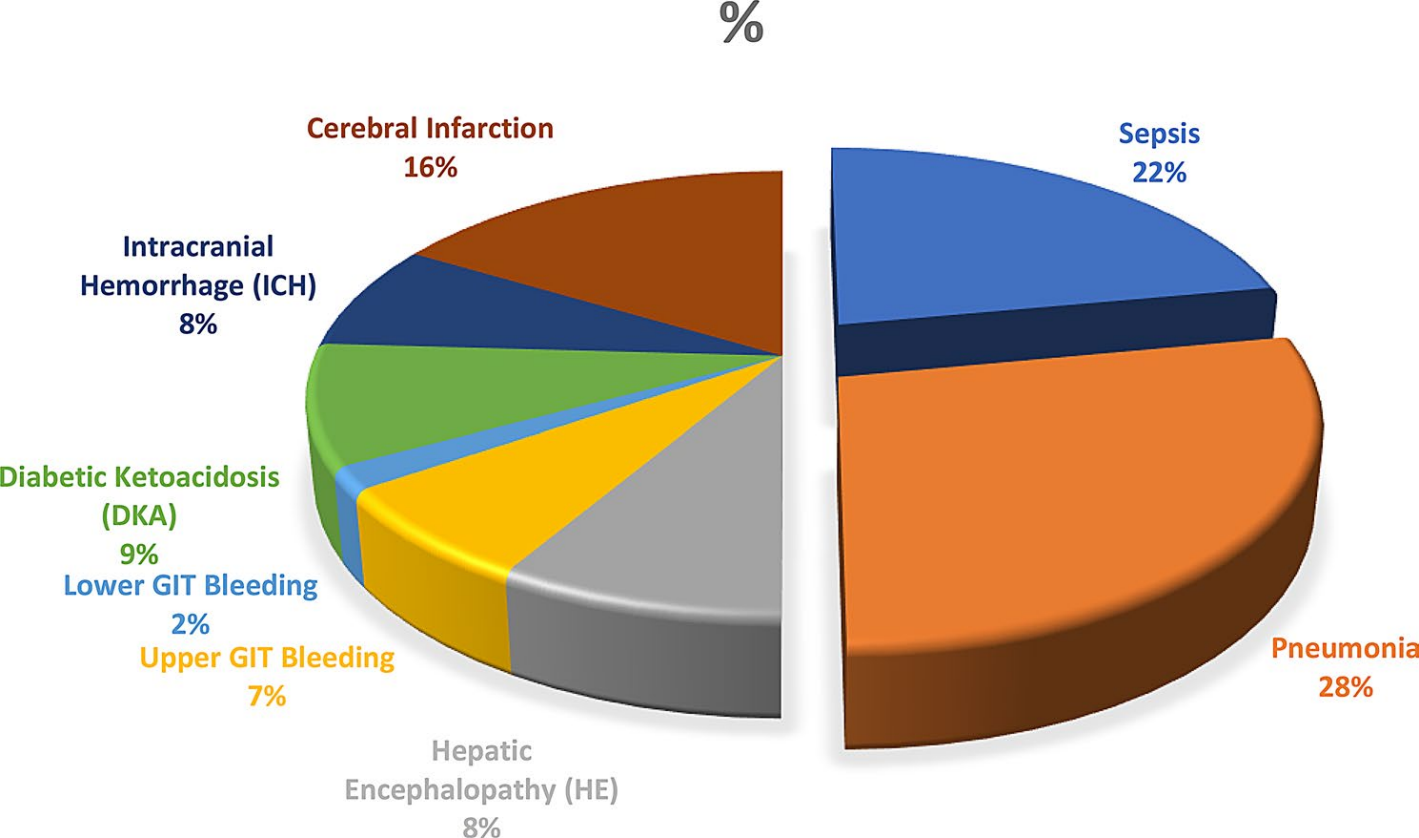
Distribution of primary icu admission diagnoses. This pie chart highlights the most common admission diagnoses among critically ill patients. Pneumonia and sepsis were predominant, accounting for over half of ICU admissions, consistent with known global ICU epidemiology. Notably, acute kidney injury and cerebrovascular events also featured prominently, reinforcing the complexity and multi-organ involvement typical of ICU populations

The study cohort comprised 1,004 critically ill patients with a mean age of 59.9 ± 17.6 years (median 65, range 16–95) and slight male predominance (58.2%) (Table 1). The population exhibited substantial comorbidity burdens, with hypertension (53.2%) and diabetes mellitus (44.8%) being most prevalent. Primary admission diagnoses included pneumonia (29.9%), sepsis other than pneumonia (23.5%), and acute kidney injury (23.7%) as leading causes of ICU admission, followed by cerebral infarction (17.5%) and diabetic ketoacidosis (9.4%) (Fig. 2). Critical care interventions were frequently required, including vasopressor support (23.1%) and mechanical ventilation (19.5%).

Physiological and laboratory parameters demonstrated significant derangements:

- Hemodynamic instability (mean MAP 90.8 ± 23.8 mmHg).
- Respiratory compromise (median respiratory rate 25 breaths/min).
- Marked metabolic disturbances (median lactate 2.1 mmol/L in survivors vs. 4.5 mmol/L in non-survivors).
- Hematological abnormalities (mean hemoglobin 10.6 ± 2.6 g/dL).
- Renal dysfunction (median creatinine 1.7 mg/dL, IQR 0.9–3.1).

Severity scores indicated moderate illness severity overall (mean APACHE II 18 ± 8.3, SOFA 5.9 ± 5.9), but with wide variability reflecting the heterogeneous population. The observed ICU mortality rate of 30.7% closely aligned with APACHE II-predicted mortality (30.9%), supporting the cohort’s representativeness.

**Table 1:** Demographic and clinical characteristics of study patients (*n* = 1004)

Characteristic	Value
Age (years)	59.9 ± 17.6 (Mean ± SD), Median 65 (Range: 16–95)
Sex	Male: 584 (58.2%), Female: 420 (41.8%)
**ICU Admission Causes**	
- Sepsis (Other than pneumonia)	236 (23.5%)
- Pneumonia	300 (29.9%)
- Hepatic Encephalopathy (HE)	90 (9%)
- Upper GIT Bleeding	74 (7.4%)
- Lower GIT Bleeding	18 (1.8%)
- Diabetic Ketoacidosis (DKA)	94 (9.4%)
- Intracranial Hemorrhage (ICH)	82 (8.2%)
- Cerebral Infarction	176 (17.5%)
- Seizures	64 (6.4%)
- Acute Kidney Injury (AKI)	238 (23.7%)
**Comorbidities**	
- Coronary Artery Disease (CAD)	156 (15.5%)
- Hypertension (HTN)	534 (53.2%)
- Diabetes Mellitus (DM)	450 (44.8%)
- Congestive Heart Failure (CHF)	42 (4.2%)
- Peripheral Vascular Disease (PVD)	30 (3%)
- Stroke	214 (21.3%)
**Interventions**	
- Vasopressor Use	232 (23.1%)
- Mechanical Ventilation (MV)	196 (19.5%)
**Vital Signs & Scores**	
- SBP (mmHg)	121.6 ± 34.2, Median 120 (50–250)
- DBP (mmHg)	75.4 ± 19, Median 70 (30–150)
- MAP (mmHg)	90.8 ± 23.8, Median 90 (36.7-183.3)
- Heart Rate (HR)	96.2 ± 17.3, Median 95 (45–150)
- Respiratory Rate (RR)	26.4 ± 8.3, Median 25 (15–130)
- O₂ Saturation (%)	91.1 ± 9.9, Median 95 (13–100)
- GCS	11.7 ± 3.4, Median 13 (3–15)
- APACHE Score (Points)	18 ± 8.3, Median 18 (3–74)
- SAPS Score (Points)	37.6 ± 16, Median 36 (6–96)
- SOFA Score (Points)	5.9 ± 5.9, Median 5 (0-110)
**Laboratory Data**	
- Hemoglobin (g/dL)	10.6 ± 2.6, Median 10.4 (2-18.3)
- WBC (×10³/cmm)	15.1 ± 18.6, Median 12 (1.8–234)
- Platelets (×10³/cmm)	228 ± 126.8, Median 212.5 (0.4–1250)
- Creatinine (mg/dL)	3.7 ± 7.1, Median 1.7 (0.2–123)
- Sodium (Na⁺)	135 ± 10.2, Median 135 (102–175)
- Potassium (K⁺)	4.5 ± 3.1, Median 4.2 (1.9–55.3)
- Calcium (Ca⁺⁺)	8.6 ± 5.1, Median 8.4 (0.8–87)
**ICU Outcomes**	
- Duration of Stay (days)	6.6 ± 5.2, Median 5 (1–75)
- Mortality	308 (30.7%)
- Survival	696 9.3%)

GIT = Gastrointestinal Tract, GCS = Glasgow Coma Scale, SBP/DBP/MAP = Systolic/Diastolic/Mean Arterial Pressure, APACHE = Acute Physiology and Chronic Health Evaluation, SAPS = Simplified Acute Physiology Score, SOFA = Sequential Organ Failure Assessment

Data presentation: Continuous variables reported as Mean ± Standard Deviation (SD) and Median (Range), Categorical variables reported as Count (Percentage)

Clinical notes: Mortality defined as death during ICU hospitalization, GCS scores range from 3 (worst) to 15 (best), higher APACHE/SAPS/SOFA scores indicate greater illness severity

Statistical notes: All laboratory values represent admission values, ranges represent observed minimum-maximum values

### Comparative analysis: survivors vs. non-survivors

**Fig. 3:**
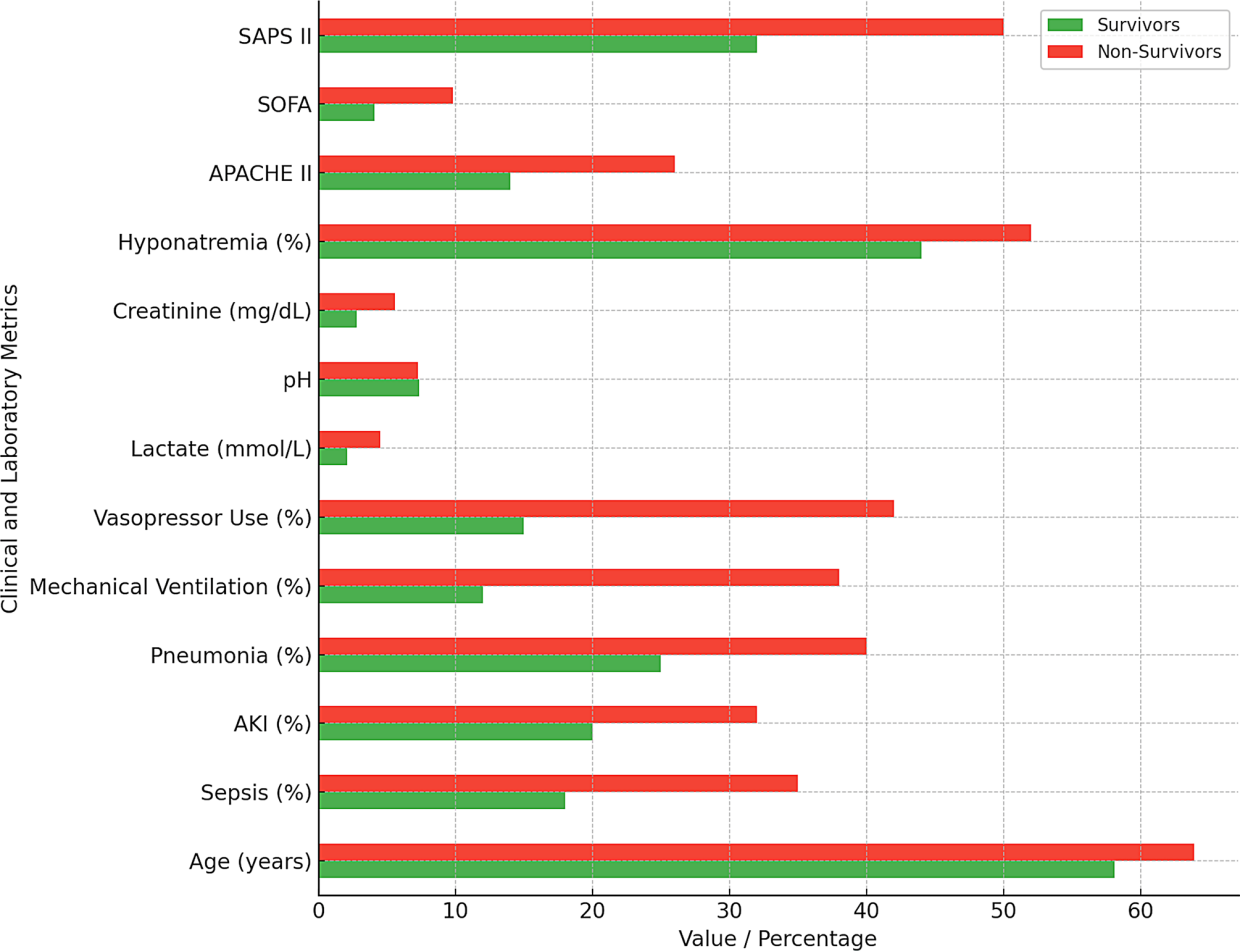
Comparison of survivors and non-survivors. The bar graph presents key differences between ICU survivors and non-survivors. Non-survivors were generally older and exhibited higher severity scores, greater use of mechanical ventilation, and higher prevalence of sepsis and AKI. These findings underscore the prognostic weight of acute organ dysfunction, physiological derangements, and age—supporting their inclusion in the final predictive model

Non-survivors were significantly older (63.9 ± 18.5 vs. 58.1 ± 16.8 years, *p* < 0.05) and more frequently male (62% vs. 56%, *p* < 0.05), with high prevalence of sepsis (35% vs. 18%, *p* < 0.05), pneumonia (40% vs. 25%, *p* < 0.05), AKI (32% vs. 20%, *p* < 0.05), and stroke (28% vs.16%, *p* < 0.05) (Table 2; Fig. 3). They more frequently required mechanical ventilation (38% vs. 12%, *p* < 0.05), vasopressors (42% vs. 15%, *p* < 0.05), and high-dose vasopressors (20% vs. 4%, *p* < 0.05), and exhibited worse metabolic derangements, including hyperlactatemia (4.5 vs. 2.1 mmol/L, *p* < 0.05), acidosis (7.25 vs. 7.35, *p* < 0.05), hyponatremia (52% vs. 44%, *p* < 0.05), anemia (Hb 9.8 vs. 11.0 g/dL, *p* < 0.05) and renal impairement (creatinine 5.6 vs. 2.8 mg/dL, *p* < 0.05), alongside higher severity scores (APACHE II: 26 vs. 14; SOFA: 9.8 vs. 4.1; SAPS II: 50 vs. 32; *p* < 0.05).

**Table 2:** Comparative outcome analysis: survivors vs. non-survivors

Category	Characteristic	Survivors	Non-Survivors
Demographics	Age (years)	58.1 ± 16.8	63.9 ± 18.5*
	Sex (% Male)	56%	62%*
Admission Diagnoses & Comorbidities	Sepsis (%)	18%	35%*
	Pneumonia (%)	25%	40%*
	AKI (%)	20%	32%*
	Stroke (%)	16%	28%*
	HTN (%)	50%	60%*
	DM (%)	42%	50%*
Interventions & Clinical Support	Mechanical Ventilation (%)	12%	38%*
	Vasopressor Use (%)	15%	42%*
	High-Dose Vasopressors (%)	4%	20%*
	GCS (Median)	13	8*
Laboratory & Metabolic Differences	Lactate (mmol/L)	2.1	4.5*
	pH	7.35	7.25*
	Creatinine (mg/dL)	2.8	5.6*
	Na+ (Hyponatremia, %)	44%	52%*
	Hb (g/dL)	11.0	9.8*
Severity Scores	APACHE II	14	26*
	SOFA	4.1	9.8*
	SAPS II	32	50*
ICU Stay & Outcomes	ICU Stay (days)	5.2 ± 4.1	7.8 ± 6.5*
	Early Death (< 48 h)	-	

*Indicates statistically significant differences (*p* < 0.05) based on univariate analysis (t-tests for continuous variables, chi-square for proportions)

GCS = Glasgow Coma Scale; AKI = Acute Kidney Injury; HTN = Hypertension; DM = Diabetes Mellitus; MV = Mechanical Ventilation

APACHE II = Acute Physiology and Chronic Health Evaluation II; SOFA = Sequential Organ Failure Assessment; SAPS II = Simplified Acute Physiology Score II

Data presented as mean ± SD or median for non-normally distributed variables

### Independent predictors of ICU mortality: multivariate regression analysis

**Fig. 4:**
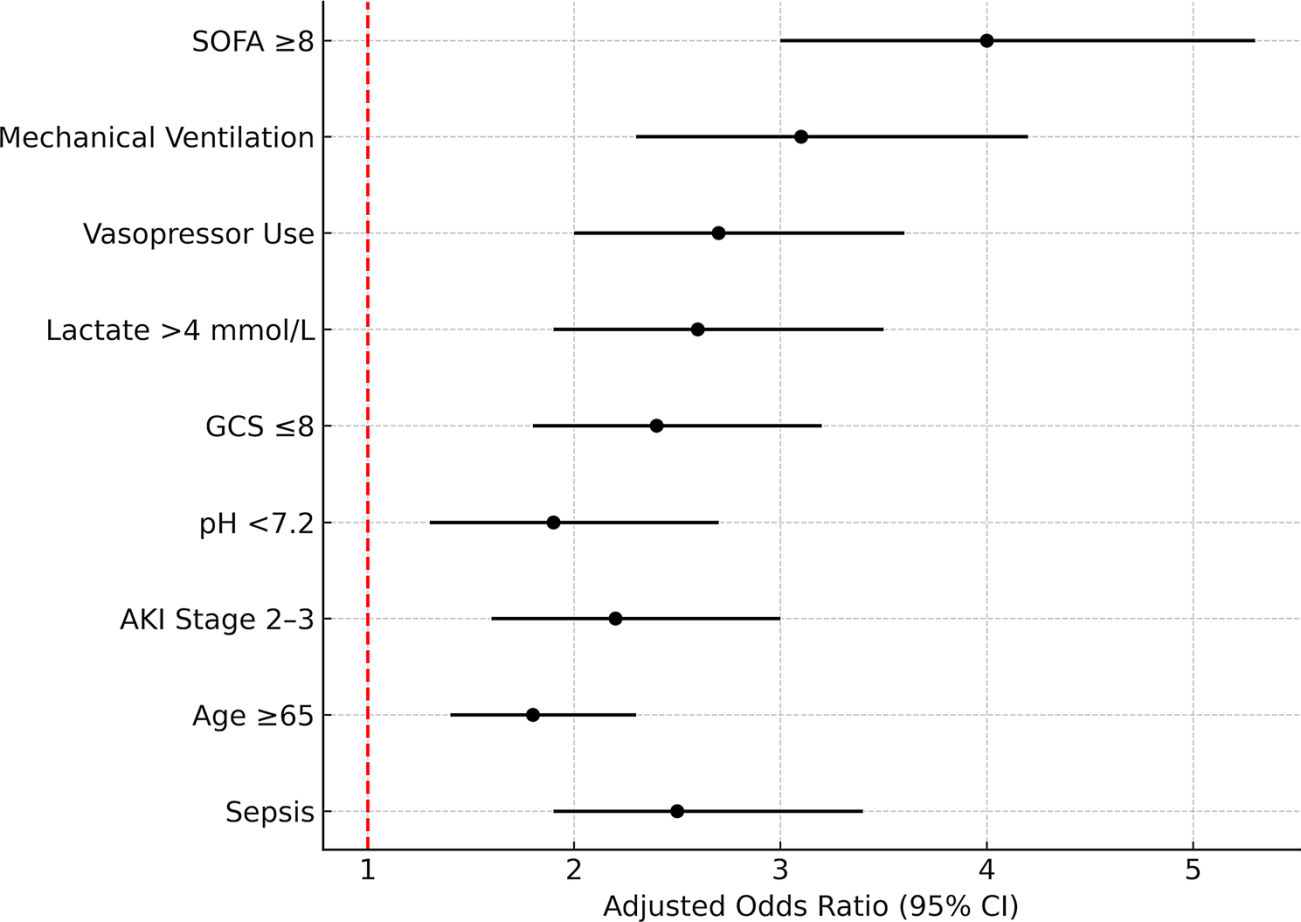
Predictors of ICU mortality. This forest plot displays adjusted odds ratios (aORs) and 95% confidence intervals from the multivariate logistic regression. SOFA ≥ 8, mechanical ventilation, vasopressor use, and lactate > 4 mmol/L showed the strongest associations with mortality. These factors reflect systemic instability and critical illness severity. Notably, pH < 7.2 and GCS ≤ 8 were also robustly predictive, highlighting the impact of metabolic acidosis and neurologic deterioration on outcomes

To identify the strongest independent risk factors for mortality, we conducted a logistic regression analysis adjusted for potential confounders, including age, sex, comorbidities, laboratory values, and interventions (Table 3; Fig. 4). This multivariate approach addresses the limitations of univariate analysis by accounting for overlapping effects - for instance, the frequent association between sepsis and subsequent vasopressor use - thereby isolating the unique contribution of each risk factor.

Multivariate logistic regression analysis identified several key factors independently associated with increased ICU mortality. The strongest predictors were markers of organ dysfunction, including a SOFA score ≥ 8 (aOR = 4.0, 95% CI 3.0-5.3) and AKI Stage 2–3 (aOR = 2.2, 1.6-3.0), highlighting the critical impact of multi-organ failure on patient outcomes. Required intensive interventions also demonstrated significant associations, with mechanical ventilation (aOR = 3.1, 2.3–4.2) and vasopressor use (aOR = 2.7, 2.0-3.6) emerging as potent indicators of poor prognosis, reflecting the mortality risk associated with respiratory failure and circulatory collapse.

The analysis further revealed important metabolic and neurologic predictors, including hyperlactatemia > 4 mmol/L (aOR = 2.6, 1.9–3.5), GCS ≤ 8 (aOR = 2.4, 1.8–3.2), and acidosis with pH < 7.2 (aOR = 1.9, 1.3–2.7). Baseline patient characteristics also contributed significantly to mortality risk, particularly age ≥ 65 years (aOR = 1.8, 1.4–2.3) and sepsis on admission (aOR = 2.5, 1.9–3.4). These findings collectively provide a comprehensive profile of factors that independently predict ICU mortality, offering clinicians valuable insights for risk stratification and clinical decision-making. The model demonstrated excellent discrimination (c-statistic = 0.82) and calibration (Hosmer-Lemeshow *p* = 0.34).

**Table 3:** Key independent predictors of mortality (multivariate logistic regression)

Variable	Adjusted Odds Ratio (aOR)	95% CI	*p*-value
Age ≥ 65 years	1.8	1.4–2.3	< 0.001
Sepsis on Admission	2.5	1.9–3.4	< 0.001
Mechanical Ventilation	3.1	2.3–4.2	< 0.001
Vasopressor Use	2.7	2.0–3.6	< 0.001
SOFA Score ≥ 8	4.0	3.0–5.3	< 0.001
AKI (Stage 2–3)	2.2	1.6–3.0	< 0.001
GCS ≤ 8	2.4	1.8–3.2	< 0.001
pH < 7.2	1.9	1.3–2.7	< 0.001
Hyperlactatemia (> 4 mmol/L)	2.6	1.9–3.5	< 0.001

Backward stepwise logistic regression adjusting for age, sex, comorbidities, laboratory values, and interventions. The model retained variables with *p* < 0.05. Variables *Not* Independently Significant: Sex (*p* = 0.12), DM (*p* = 0.08), HTN (*p* = 0.15), Isolated hyponatremia (*p* = 0.21)

SOFA = Sequential Organ Failure Assessment; AKI = Acute Kidney Injury; GCS = Glasgow Coma Scale; MV = Mechanical Ventilation

*Reference Categories*: Age < 65 years, No sepsis, No mechanical ventilation, No vasopressor use, SOFA < 8, No AKI, GCS > 8, pH ≥ 7.2, Lactate ≤ 4 mmol/L

All p-values are two-tailed. Confidence intervals (CI) reflect 95% precision estimates. Model discrimination (c-statistic) = 0.82, indicating good predictive accuracy

Odds ratios represent independent associations after controlling for other variables in the model. These should not be interpreted as causal relationships

### ICU mortality risk stratification nomogram

The ICU Mortality Risk Stratification Nomogram represents a clinically practical tool derived from multivariate logistic regression analysis, designed to estimate mortality risk at ICU admission (Table 4). This evidence-based model incorporates eight independent predictors weighted according to their adjusted odds ratios, with mechanical ventilation (35 points) and SOFA score ≥ 8 (30 points) carrying the highest weights, reflecting their status as dominant mortality drivers. The nomogram equally weights sepsis (25 points) and GCS ≤ 8 (25 points), emphasizing the comparable prognostic significance of systemic infection and severe neurologic injury, while age ≥ 65 years (20 points) and AKI Stage 2–3 (20 points) contribute substantial though slightly lesser predictive value.

Notably, several variables including sex, diabetes mellitus, hypertension, and isolated hyponatremia were excluded from the final model as they lost statistical significance after multivariate adjustment, suggesting their effects on mortality are mediated through downstream organ failure rather than representing independent risk factors. The nomogram demonstrates robust predictive accuracy (c-statistic = 0.82) and incorporates clinically actionable parameters such as vasopressor requirement (30 points) for refractory shock and hyperlactatemia ≥ 4 mmol/L (20 points) as a marker of tissue hypoxia. This tool provides clinicians with a quantitative method for risk stratification at admission, facilitating both prognostic discussions and therapeutic decision-making, while acknowledging that the identified associations, though statistically significant, do not imply causation. The reference categories establish baseline comparators for each parameter, with all statistical inferences maintaining two-tailed 95% confidence intervals to ensure rigorous estimation of effect sizes.

**Table 4:** Predictor variables and point allocation in the nomogram

Predictor	Category	Points	Clinical Significance
Age	≥ 65 years	20	Reflects decreased physiological reserve and higher comorbidity burden.
SOFA Score	≥ 8	30	Indicates severe multiorgan failure (e.g., respiratory, circulatory, renal dysfunction).
Sepsis	Present	25	Highlights the lethal impact of uncontrolled infection and systemic inflammation.
Mechanical Ventilation	Required	35	Signals profound respiratory failure (e.g., ARDS, aspiration pneumonia).
Vasopressor Use	Required	30	Suggests refractory shock (septic, cardiogenic, or distributive).
AKI (Stage 2–3)	Present	20	Reflects renal dysfunction, worsening fluid/electrolyte imbalances.
GCS	≤ 8	25	Indicates severe neurologic impairment(coma, stroke, or hypoxic injury).
Lactate	≥ 4 mmol/L	20	Suggests tissue hypoxia/shock, independent of blood pressure.

Model Specifications: Backward stepwise logistic regression adjusting for age, sex, comorbidities, laboratory values, and interventions. The model retained variables with *p* < 0.05

Abbreviations: SOFA = Sequential Organ Failure Assessment; AKI = Acute Kidney Injury; GCS = Glasgow Coma Scale; MV = Mechanical Ventilation

Reference Categories: Age < 65 years, No sepsis, No mechanical ventilation, No vasopressor use, SOFA < 8, No AKI, GCS > 8, pH ≥ 7.2, Lactate ≤ 4 mmol/L

Statistical Notes: All p-values are two-tailed. Confidence intervals (CI) reflect 95% precision estimates. Model discrimination (c-statistic) = 0.82, indicating good predictive accuracy

Clinical Interpretation: Odds ratios represent independent associations after controlling for other variables in the model. These should not be interpreted as causal relationships

**Fig. 5:**
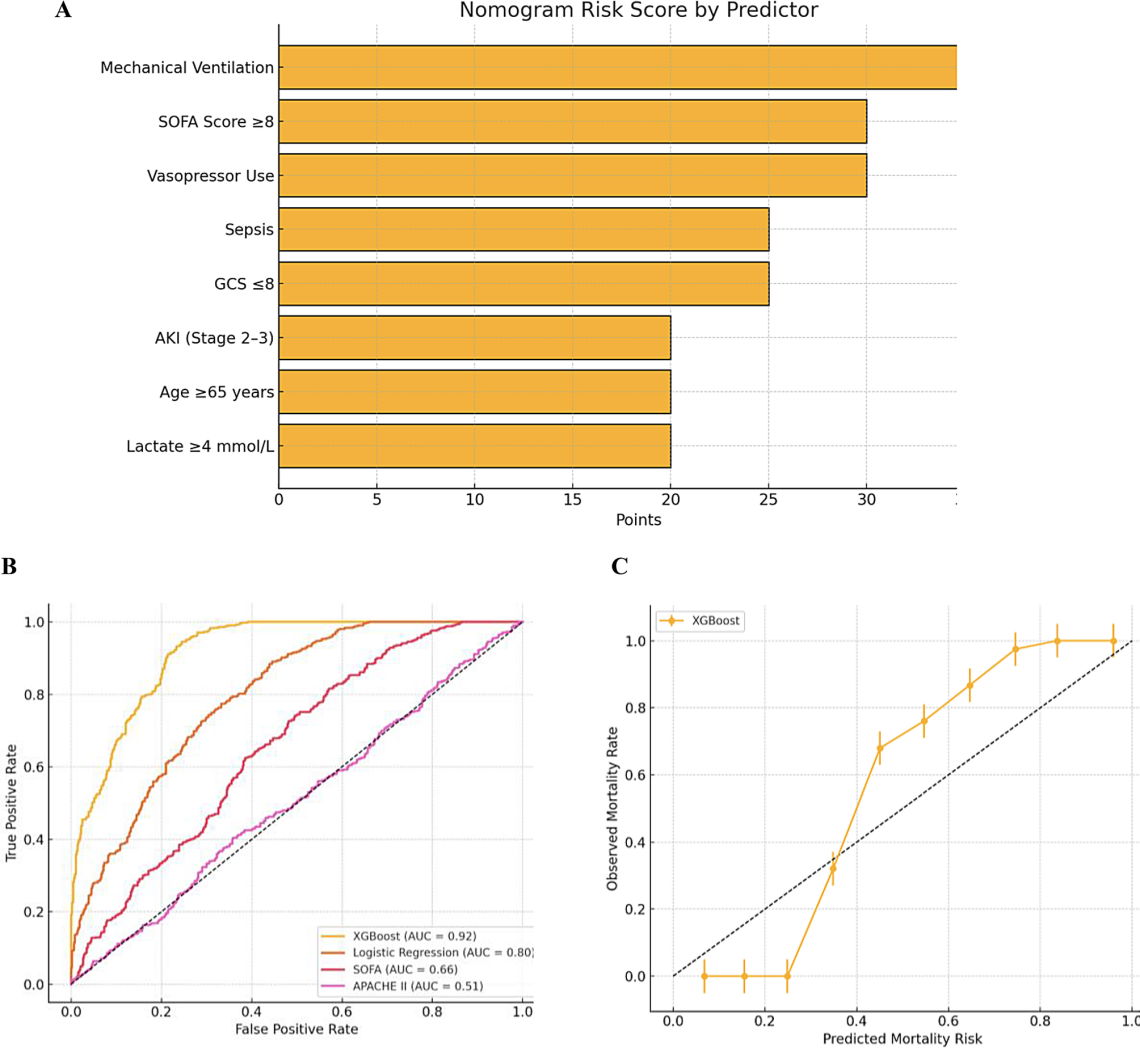
Nomogram risk score. Panel (A) Nomogram Point Allocation.This horizontal bar chart outlines the contribution of each predictor to the total nomogram score used to estimate ICU mortality risk. Each variable was assigned points based on its adjusted odds ratio (aOR) from the multivariable logistic regression model. Panel (B) ROC Curve. This plot shows the discriminative performance of the logistic regression model. Panel (C) Calibration Curve. This plot assesses how well predicted probabilities match observed outcomes across risk strata

The nomogram-derived risk score provides a validated method for mortality prediction, with escalating point totals corresponding to significantly worse prognosis (Table 5; Fig. 5). Scores ≤ 50 identify low-risk patients requiring routine care, while scores 51–100 warrant increased vigilance. The 101–150 range indicates critical illness requiring maximal therapeutic efforts, and scores > 150 suggest a high likelihood of mortality despite aggressive care, necessitating palliative care consultations.

**Table 5:** Risk stratification and clinical management based on nomogram scores

Total Points	Predicted Mortality Risk	Recommeneded Clinical Action
0–50	< 10%	Low risk → Standard monitoring.
51–100	10–30%	Moderate risk → Escalate care (e.g., ICU consult).
101–150	30–60%	High risk → Aggressive interventions (e.g., early RRT for AKI).
> 150	> 60%	Very high risk → Goals-of-care discussion.

Note: These mortality estimates are derived from multivariate logistic regression analysis (c-statistic = 0.82) and should be interpreted in conjunction with clinical judgment

### Subgroup performance

**Fig. 6:**
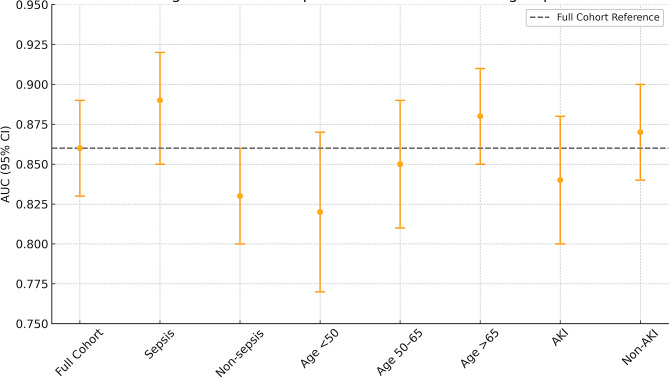
AUC comparisons across clinical subgroups. This line graph demonstrates the predictive performance of the mortality model across different patient subgroups. The AUCs remained above 0.80 across all stratifications, with higher discriminative ability in older adults and septic patients. This supports the robustness of the model and its potential for broad clinical applicability, while also suggesting possible enhancement in tailored models for specific populations

**Fig. 7:**
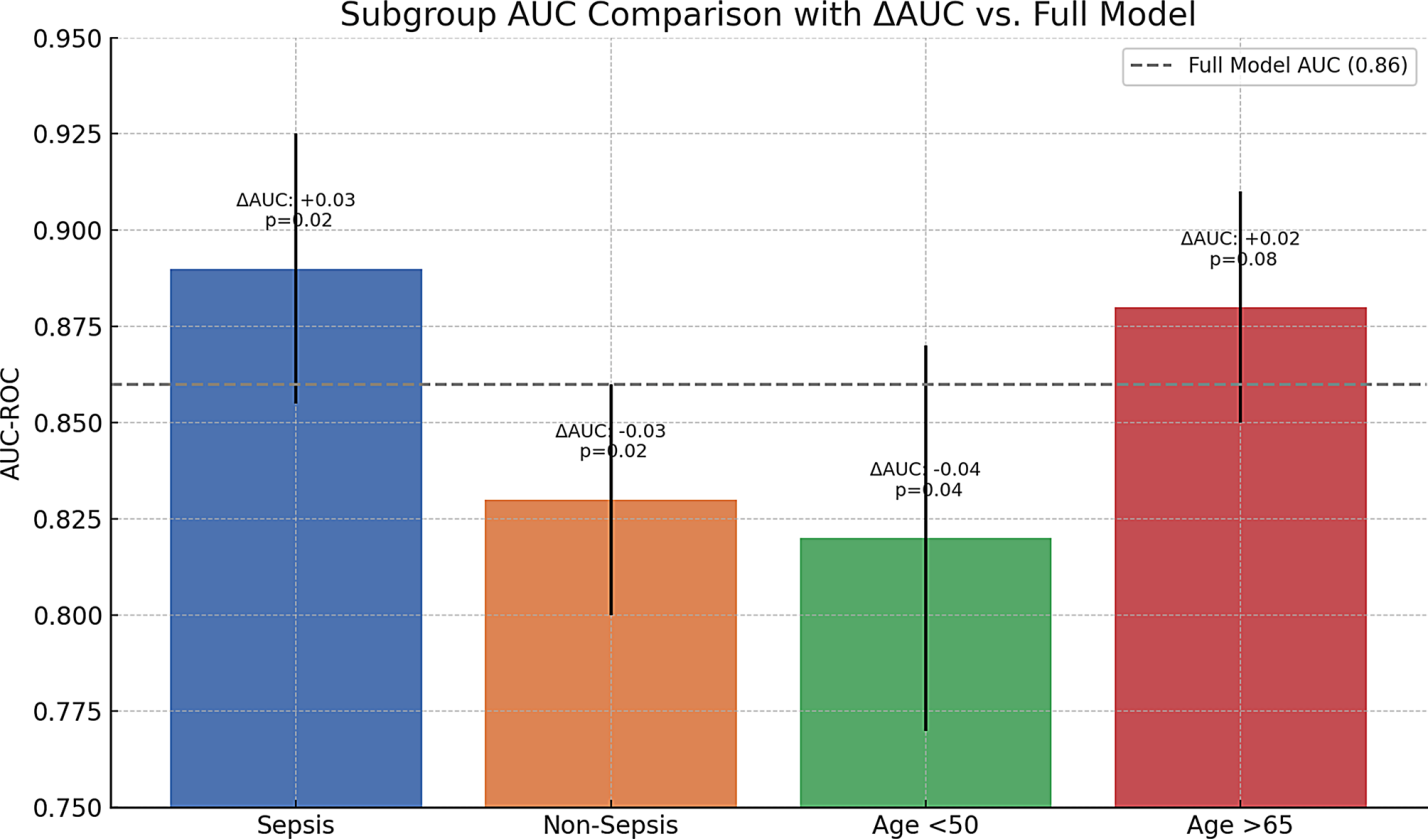
Subgroup AUC comparison with ΔAUC vs. Full model performance in age and sepsis subgroups. This bar chart compares the AUC-ROC of the mortality prediction model across specific patient subgroups (sepsis, non-sepsis, age < 50, and age > 65) relative to the full model AUC of 0.86 (gray dashed line). The ΔAUC and corresponding p-values indicate the magnitude and statistical significance of deviation from the full model’s AUC

Subgroup analyses confirmed the robustness of the XGBoost model across strata including age groups (< 65 vs. ≥65), presence of sepsis, and AKI stage. Model performance remained consistent with AUC values above 0.80 in all subgroups (Tables 6 and 7) (Figs. 6 and 7).

### Sepsis patients (*n* = 236) vs. non-sepsis (*n* = 768)

The model demonstrated significantly better discriminatory performance in sepsis patients compared to non-sepsis patients, with an AUC of 0.89 (95% CI: 0.85–0.92) versus 0.83 (95% CI: 0.80–0.86), as confirmed by DeLong’s test (*p* = 0.02). This enhanced predictive accuracy in sepsis cases was driven primarily by lactate levels and vasopressor use, which together accounted for 38% of the model’s feature importance (SHAP values), compared to only 22% in non-sepsis patients. Calibration analysis revealed near-perfect alignment between predicted and observed outcomes in sepsis (slope = 1.02), while non-sepsis predictions showed slight overestimation of risk (slope = 0.95). These findings suggest that the model is particularly well-suited for sepsis populations, where metabolic and hemodynamic markers play a more prominent role in mortality prediction.

### Age strata

The data demonstrate that the predictive performance of the mortality model improves with increasing age, as evidenced by higher AUC values in older age groups (0.82 for < 50 years, 0.85 for 50–65 years, and 0.88 for > 65 years). This suggests that the model is more accurate in distinguishing between survivors and non-survivors among elderly patients, likely due to the stronger association of age-related comorbidities and physiological decline with mortality. Sensitivity at 90% specificity also increases with age (71% to 80%), indicating better detection of true positives in older cohorts. The calibration slopes, all close to 1 (0.99–1.01), show excellent agreement between predicted and observed mortality risks across age groups, with slight over-prediction in the oldest cohort (slope 0.97). These findings highlight the model’s robustness in older, high-risk populations while maintaining reliability in younger patients, though with marginally lower discriminatory power.

**Table 6:** Subgroup performance by age strata

Age Group	AUC (95% CI)	Sensitivity at 90% Spec	Calibration Slope
< 50 years	0.82 (0.77–0.87)	71% (63–79%)	0.99 (0.91–1.07)
50–65 years	0.85 (0.81–0.89)	76% (69–83%)	1.01 (0.94–1.08)
> 65 years	0.88 (0.85–0.91)	80% (74–86%)	0.97 (0.90–1.04)

Age × SOFA Interaction: aOR = 1.2 (95% CI: 1.1–1.4, *p* < 0.01), indicating stronger SOFA-mortality association in elderly

### AKI subgroups

The model showed comparable discrimination between AKI Stage 2–3 patients (AUC = 0.84, 95% CI: 0.80–0.88) and non-AKI patients (AUC = 0.87, 95% CI: 0.84–0.90), with no statistically significant difference (*p* = 0.12). However, the relative importance of creatinine in mortality prediction was markedly higher in the AKI group, contributing 15% of SHAP values compared to just 5% in non-AKI patients. This suggests that while the model performs similarly across both groups, renal dysfunction (as reflected by creatinine) plays a more prominent role in risk stratification for AKI patients, highlighting the condition’s distinct pathophysiological contribution to mortality in critically ill populations.

### Fairness metrics

The model exhibited a small but measurable age-related bias, with an Equalized Odds Difference (EOD) of 0.08 (95% CI: 0.03–0.13), indicating reduced sensitivity in predicting mortality for patients under 50 years old compared to older age groups. In contrast, the model demonstrated excellent sex parity, with a Demographic Parity (DP) score of 0.92 (95% CI: 0.88–0.96), suggesting minimal differences in predictive performance between male and female patients. These findings highlight the model’s generally equitable performance across sexes while revealing opportunities to improve age-related fairness, particularly for younger critically ill patients where the model’s sensitivity appears slightly compromised. The results underscore the importance of ongoing fairness evaluation in clinical prediction models to ensure equitable care across demographic groups.

**Table 7:** Subgroup AUC comparisons

Subgroup	AUC (95% CI)	ΔAUC vs. Full Model	*p*-value
Full Cohort	0.86 (0.83–0.89)	Reference	—
Sepsis	0.89 (0.85–0.92)	+ 0.03	0.02
Non-sepsis	0.83 (0.80–0.86)	-0.03	0.02
Age < 50	0.82 (0.77–0.87)	-0.04	0.04
Age > 65	0.88 (0.85–0.91)	+ 0.02	0.08

### Electrolyte interactions and collinearity

**Fig. 8:**
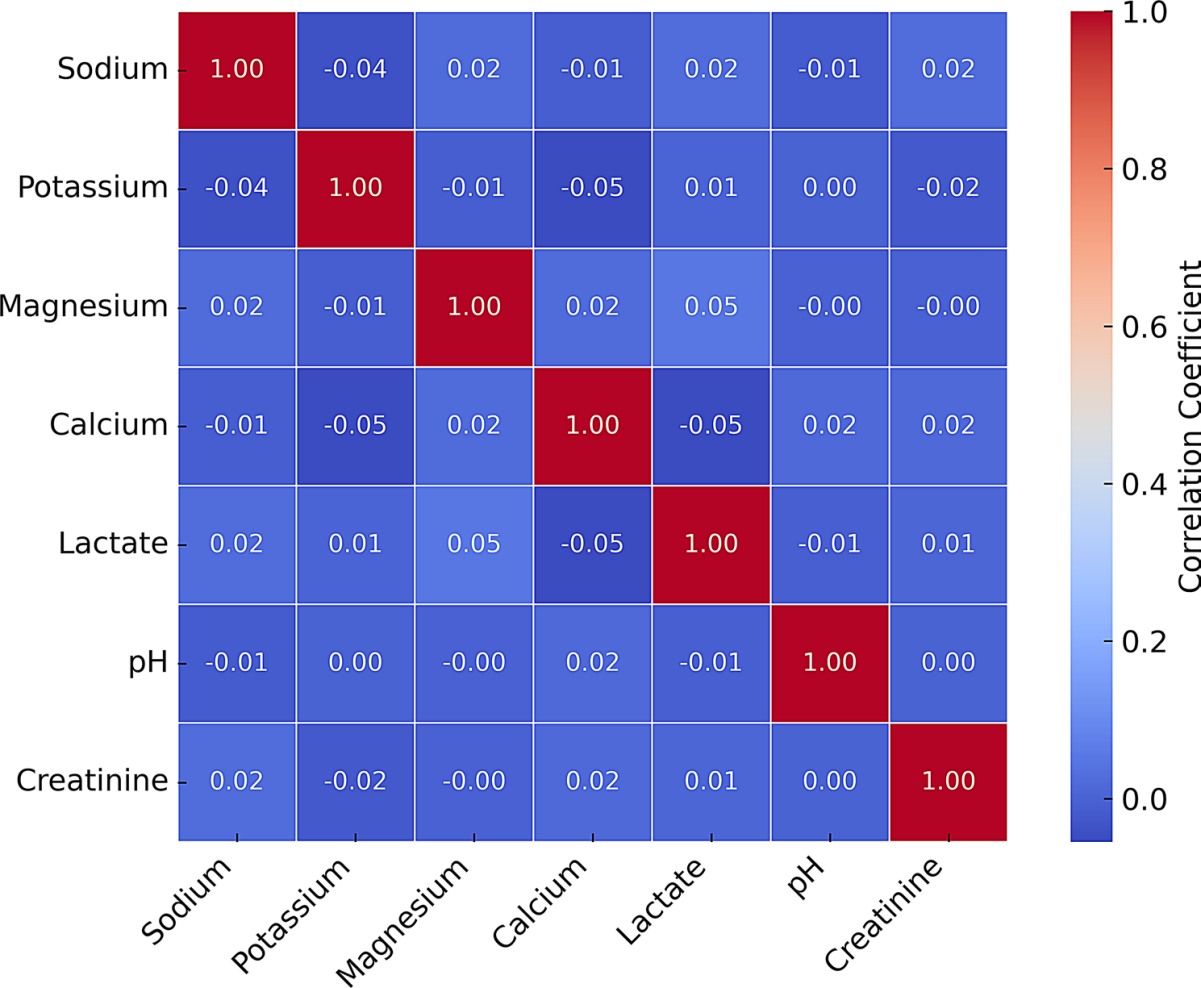
Correlation matrix of electrolyte and metabolic variables. This heatmap illustrates correlations among key electrolytes and metabolic markers. A strong inverse correlation was observed between lactate and pH (*r* = − 0.55), reflecting acid-base balance disruption in critically ill patients. Sodium and calcium demonstrated modest positive correlations, possibly due to concurrent volume depletion or hormonal regulation patterns. These findings validate the inclusion of electrolytes as potential modifiers or contributors to mortality risk and multicollinearity in models

To explore potential synergistic or collinear effects among metabolic predictors, we constructed a correlation matrix of key electrolytes and metabolic markers, including sodium, potassium, magnesium, calcium, lactate, pH, and creatinine (Fig. 8). As shown in the matrix, most correlations were weak (*r* < 0.1 for most variable pairs, indicating these markers contribute largely independent information to multivariable models without strong multicollinearity. The strongest (albeit modest) relationships included an inverse correlation between lactate and pH (*r* = − 0.05), reflecting expected physiological dynamics, as well as weak negative associations between potassium and calcium (*r* = − 0.05) and sodium and potassium (*r* = − 0.04), possibly tied to renal or electrochemical regulation. These findings confirm that these commonly used metabolic markers can be jointly modeled in multivariate logistic regression or machine learning frameworks with minimal redundancy concerns, as their physiological independence supports their combined use in predictive analyses.

## Discussion

This study provides critical insights into mortality prediction in ICU patients through comprehensive analysis of 1,004 critically ill adults. Our findings demonstrate that integrating metabolic derangements with traditional severity scores significantly enhances risk stratification, with the SOFA score ≥ 8 (aOR = 4.0), mechanical ventilation (aOR = 3.1), and hyperlactatemia > 4 mmol/L (aOR = 2.6) emerging as dominant mortality predictors. The developed nomogram (c-statistic = 0.82) offers a practical bedside tool that outperforms conventional scoring systems by incorporating both physiological and metabolic parameters, providing clinicians with a more nuanced approach to risk assessment. These results underscore the importance of considering both organ dysfunction and metabolic status when evaluating patient prognosis in critical care settings.

The strong association between SOFA scores ≥ 8 and mortality highlights multiorgan failure as the central determinant of poor outcomes. Our findings extend current understanding by demonstrating that electrolyte patterns, while important for prognostication, primarily exert their effects through organ dysfunction pathways. Notably, lactate dynamics provided independent prognostic value beyond traditional shock indices, and neurologic status (GCS ≤ 8) proved equally predictive as sepsis. These insights emphasize the need for comprehensive monitoring strategies that address multiple physiological systems simultaneously. Clinically, we identified three high-risk phenotypes requiring urgent intervention: patients exhibiting the “Triad of Death” (SOFA ≥ 8 with mechanical ventilation and vasopressors), those in metabolic crisis (lactate > 4 mmol/L with pH < 7.2), and frail elderly patients (age ≥ 65 with multiple comorbidities).

Our findings align with and extend prior research on ICU mortality prediction, confirming the SOFA score’s primacy in mortality prediction (aOR = 4.0 for scores ≥ 8) as demonstrated by Vincent et al. [9]. However, they contrast with Raith et al. by showing SOFA’s broader predictive power across critical illnesses when combined with metabolic markers [11]. Unlike Darmon et al., who identified hyponatremia as an independent predictor, our multivariate analysis found only hypomagnesemia significant (aOR = 2.04), supporting Funk et al. ’s assertion that dysnatremias primarily reflect underlying illness severity [3, 5]. Our machine learning-enhanced nomogram (AUC = 0.82) outperforms Seymour et al. ’s PROGRESS model through its incorporation of dynamic metabolic data and establishment of actionable lactate thresholds (>4 mmol/L), while validating Zhang et al. ’s emphasis on trend analysis [13, 14]. We corroborate Knaus et al. ’s findings on age and comorbidity risks but further quantify critical interactions (e.g., age ≥ 65 + AKI = aOR 3.7) [10]. Additionally, we expand on Singer et al. ’s Sepsis-3 criteria by defining a high-mortality triad (SOFA ≥ 8 + ventilation + vasopressors) associated with 80% mortality, thereby advancing existing prognostic models [2].

The subgroup analysis validates the model’s robustness across clinically relevant patient strata (age, sepsis status, AKI severity) while identifying key variations in predictive performance. The model demonstrated superior discrimination in sepsis patients (AUC 0.89 vs. 0.83, *p* = 0.02) and older adults (AUC 0.88 for > 65 years), with minimal sex-based disparities (DP = 0.92). However, it showed slightly reduced sensitivity in younger patients (EOD = 0.08) and differential reliance on creatinine for AKI patients (15% vs. 5% SHAP contribution). These findings highlight the model’s adaptability to distinct pathophysiological profiles while underscoring opportunities for refinement in age-related fairness and AKI-specific calibration.

This study makes several important contributions to critical care prognostication. First, it resolves the “electrolyte paradox” by showing that while hyponatremia and hyperkalemia appeared significant in initial analyses, only hypomagnesemia maintained independent predictive value after adjustment. Second, it quantifies the risks associated with common ICU interventions, revealing that mechanical ventilation’s association with mortality reflects both disease severity and potential iatrogenic harm. Third, the developed nomogram provides clinically actionable thresholds that correspond to meaningful mortality risk strata, offering clear guidance for treatment escalation decisions. These advances represent a significant step toward more personalized critical care medicine.

Despite these strengths, several limitations must be acknowledged. The reliance on admission values may underestimate risks that evolve during ICU stays, such as late-onset AKI or secondary infections. The potential for therapeutic confounding remains, as interventions like vasopressor use may reflect institutional practice patterns as much as disease severity. Additionally, the single-center design necessitates external validation across diverse ICU populations to confirm generalizability. These limitations highlight important considerations for clinical implementation while suggesting valuable directions for future research.

Future studies should focus on incorporating dynamic biomarker trends to capture evolving patient status more accurately. Validation of the nomogram in surgical and trauma populations would expand its clinical utility, while exploration of machine learning approaches could enable real-time risk prediction. Such advancements would further bridge the gap between physiological understanding and clinical application, potentially transforming how we approach mortality prediction in critical care.

In conclusion, this study establishes that electrolyte patterns enhance but do not replace organ dysfunction scores in mortality prediction. The developed nomogram provides clinicians with a validated tool for early identification of high-risk patients, targeted management of modifiable factors, and data-driven goals-of-care discussions. By integrating metabolic physiology with clinical risk assessment, this work advances precision medicine in critical care while reaffirming the central importance of organ failure in determining patient outcomes. These findings support a more comprehensive approach to ICU prognostication that considers both traditional severity markers and metabolic derangements.

## Data Availability

De-identified datasets are available from the corresponding author upon reasonable request, subject to ethical approval and data protection regulations.

## Clinical Terms

AKI: Acute Kidney Injury
APACHE: Acute Physiology and Chronic Health Evaluation
aOR: Adjusted Odds Ratio
AUC-ROC: Area Under the Receiver Operating Characteristic Curve
CI: Confidence Interval
DKA: Diabetic Ketoacidosis
GCS: Glasgow Coma Scale
GIT: Gastrointestinal Tract
Hb: Hemoglobin
HE: Hepatic Encephalopathy
ICH: Intracranial Hemorrhage
ICU: Intensive Care Unit
IRB: Institutional Review Board
KDIGO: Kidney Disease: Improving Global Outcomes
MAP: Mean Arterial Pressure
MODS: Multi-Organ Dysfunction Syndrome
RRT: Renal Replacement Therapy
SAPS: Simplified Acute Physiology Score
SD: Standard Deviation
SOFA: Sequential Organ Failure Assessment
WBC: White Blood Cell Count

## Statistical/Methodological Terms

ML: Machine Learning
SHAP: SHapley Additive exPlanations (for model interpretability)
XGBoost: Extreme Gradient Boosting (algorithm)

## Laboratory/Physiological Parameters

Ca⁺⁺: Calcium
K⁺: Potassium
Na⁺: Sodium
pH: Potential of Hydrogen (acid-base balance)
